# Anesthesiology Control Tower—Feasibility Assessment to Support Translation (ACTFAST): Mixed-Methods Study of a Novel Telemedicine-Based Support System for the Operating Room

**DOI:** 10.2196/12155

**Published:** 2019-04-23

**Authors:** Teresa Murray-Torres, Aparna Casarella, Mara Bollini, Frances Wallace, Michael S Avidan, Mary C Politi

**Affiliations:** 1 Department of Anesthesiology Washington University School of Medicine in St. Louis Washington University in St. Louis St. Louis, MO United States; 2 Brown School of Social Work Washington University in St. Louis St. Louis, MO United States; 3 Department of Anesthesiology, Perioperative and Pain Medicine Brigham and Women's Hospital Boston, MA United States; 4 Department of Surgery, Division of Public Health Sciences Washington University School of Medicine in St. Louis St. Louis, MO United States

**Keywords:** clinical decision support systems, usability, anesthesiology, telemedicine

## Abstract

**Background:**

Despite efforts to improve patient outcomes, major morbidity and mortality remain common after surgery. Health information technologies that provide decision support for clinicians might improve perioperative and postoperative patient care. Evaluating the usability of these technologies and barriers to their implementation can facilitate their acceptance within health systems.

**Objective:**

This manuscript describes usability testing and refinement of an innovative telemedicine-based clinical support system, the Anesthesiology Control Tower (ACT). It also reports stakeholders’ perceptions of the barriers and facilitators to implementation of the intervention.

**Methods:**

Three phases of testing were conducted in an iterative manner. Phase 1 testing employed a think-aloud protocol analysis to identify surface-level usability problems with individual software components of the ACT and its structure. Phase 2 testing involved an extended qualitative and quantitative real-world usability analysis. Phase 3 sought to identify major barriers and facilitators to implementation of the ACT through semistructured interviews with key stakeholders.

**Results:**

Phase 1 and phase 2 usability testing sessions identified numerous usability problems with the software components of the ACT. The ACT platform was revised in seven iterations in response to these usability concerns. Initial satisfaction with the ACT, as measured by standardized instruments, was below commonly accepted cutoffs for these measures. Satisfaction improved to acceptable levels over the course of revision and testing. A number of barriers to implementation were also identified and addressed during the refinement of the ACT intervention.

**Conclusions:**

The ACT model can improve the standard of perioperative anesthesia care. Through our thorough and iterative usability testing process and stakeholder assessment of barriers and facilitators, we enhanced the acceptability of this novel technology and improved our ability to implement this innovation into routine practice.

**International Registered Report Identifier (IRRID):**

RR2-10.1186/s40814-018-0233-4

## Introduction

The last several decades have brought major advancements in the safety of anesthetic techniques and therapeutics. However, patients undergoing surgery continue to experience persistent and significant risks of major morbidity and mortality following their operations [1]. Some of these risks are unavoidable and either inherent to the nature of the surgical procedure or attributable to patient characteristics not immediately modifiable [2-4]. However, many factors that impact a patient’s immediate and long-term health can be influenced by the anesthesia care team [5-8]. Clinical decision support systems can optimize management of these factors, leading to improvement in intraoperative parameters such as hemodynamics [9], ventilator and fluid management [10], and blood glucose control [11,12]. Such systems are particularly useful for members of the anesthesia care team [13,14], who, like all medical practitioners, have known limitations in their cognitive abilities [15-17] and yet are often inundated with an overwhelming amount of information. Practitioners may see alarms as frequently as every 3 minutes and even more frequently during induction of anesthesia and emergence from anesthesia. Although the majority of alarms might appear clinically irrelevant, a small, critical percentage require immediate intervention [18]. Given the known limits of human cognitive abilities, there is a pressing need for decision support systems that improve clinicians’ abilities to rapidly assess situations and act appropriately in a timely manner [13,14].

Decision support systems provide an opportunity to impact provider behaviors and patient outcomes in a broad range of clinical settings [19-21]. However, these interventions may fail to meaningfully influence patient care if they are not acceptable to the relevant end users [22]. Successful systems are those that achieve high levels of usability [23,24] by meeting thresholds for efficiency, effectiveness, and satisfaction [25-27] in the actual environment in which they will be used [22,28,29]. However, even a usable, well-designed intervention may fail if barriers to its integration into existing workflow patterns have not been considered [29,30]. The assessment of such barriers is particularly important in the setting of clinical trials [31,32], in which the delivery of an intervention is dependent on changes in behavior across many groups of individuals working in complex systems.

At our institution, we developed a novel telemedicine-based decision support intervention for the operating room called the Anesthesiology Control Tower (ACT), described in Multimedia Appendix 1 and elsewhere [33]. In the ACT, clinicians use several electronic health records (EHRs) to monitor surgical patients in real time and respond to clinical alerts generated by a customized version of a decision support software device called AlertWatch (AlertWatch LLC). This software system, modified in response to the testing described in this manuscript to create an AlertWatch Tower Mode, is a monitoring and alerting program that integrates information from patient monitors and EHRs. After analyzing the data, the program determines and displays the current patient state and generates alerts based on the incoming variables (Multimedia Appendix 1). A key component of the ACT is the presence of trained clinicians who are able to process these alerts. Just as an air traffic control tower monitors individual aircraft and delivers additional information and alerts to the pilot and copilot, the ACT functions as a clinical support system for teams of anesthesia clinicians, engaging with them to assist in providing safe, effective, and efficient care for their patients [34]. The ACT is currently being evaluated in the form of a proof-of-principle pragmatic trial [NCT02830126] [35].

The successful execution of complex health interventions such as the ACT demands an understanding of the usability of the intervention and any barriers and facilitators to its acceptance. Therefore, we designed this study to evaluate the ACT from the perspective of two groups of key stakeholders: those who deliver and those who receive the ACT support. Specifically, we aimed to determine whether the ACT adequately addressed goals for functionality and usability for end users. We also sought to identify barriers and facilitators to implementation of the ACT into routine clinical practice. We used these findings to modify the ACT based on user feedback.

## Methods

### Study Design

Three phases of testing were designed to determine the extent to which the different aspects of the ACT prototype met the needs of end users (see Table 1). A full description of the study protocol was previously published by our group [33]. Two phases of pragmatic mixed-method usability analyses [36-40] were conducted with ACT clinicians. These phases evaluated the entirety of the ACT structure and its software components. In the third phase of testing, semistructured interviews were conducted with operating room (OR) clinicians to identify barriers and facilitators to implementation of the ACT and obtain basic usability data. The results from testing phases were intended to guide iterative changes to the ACT structure and software, in particular the AlertWatch Tower Mode platform. Decisions to modify any component of the pilot ACT during the testing period were determined by the investigative team through review of participant feedback.

**Table 1:** Description of testing stages.

Stage of testing	Description	Eligible participants^a^	Outcome measures
Phase 1	Structured think-aloud usability sessions with ACT^b^ clinicians	Attending anesthesiologists Resident anesthesiologists	Task performance data Standardized questionnaires Utterance data
Phase 2	Near-live usability testing with ACT clinicians	Attending anesthesiologists Resident anesthesiologists	Task performance data Standardized questionnaires
Phase 3	Semistructured interviews with operating room clinicians	Attending anesthesiologists Resident anesthesiologists Certified registered nurse anesthetists	Barriers and facilitators to implementation

^a^Only physician anesthesiologists were eligible for participation in phase 1 and phase 2 based on the preliminary staffing model for the ACT.

^b^ACT: Anesthesiology Control Tower.

### Participants

Participants were recruited from a single academic medical center using standardized emails distributed to the departmental listserv. All participants completed informed consent prior to study activities, consistent with the protocol approved by the Washington University in St. Louis Institutional Review Board (IRB #201611035). The target sample size for each round of testing was 8 to 10 participants based on guidelines for cognitive usability testing [41]. Based on an initial, physician-only staffing model for the ACT, only attending and resident physician anesthesiologists were eligible for participation in phase 1 and phase 2. All OR clinicians (physician anesthesiologists and certified registered nurse anesthetists [CRNAs]) were eligible to participate in phase 3.

### Study Procedures

#### Usability Testing

Phase 1 was an exploratory think-aloud usability analysis with the two groups of clinicians who were eligible to staff the ACT (ACT clinicians). It aimed to identify major surface-level usability problems with the different components of the ACT [42], including orientation and help documents prepared by the research team, individual software components, and physical equipment and layout. A research team member was present in the room to moderate each session. Participants had 20 minutes to load the AlertWatch Tower Mode software in addition to the hospital’s standard perioperative software programs, including the general EHR and the anesthesia information management system. They were instructed to address as many AlertWatch Tower Mode alerts as they could while voicing their thoughts and actions aloud [43]. Participants were prompted if 20 seconds elapsed without verbalization. If participants experienced a critical usability problem that prevented the session from continuing, the moderator provided the minimum amount of prompting that allowed the session to proceed. Sessions ended with structured debriefing sessions.

All think-aloud sessions were audio recorded and transcribed manually by a professional transcription service. Debriefing sessions were also transcribed when a recording was available. Transcripts were verified by the research team. At the end of each session, participants completed the quantitative usability and workload measures System Usability Scale (SUS) [44], Computer System Usability Questionnaire (CSUQ) [45], and NASA Task Load Index (NASA-TLX) [46].

Phase 2 consisted of usability testing of the ACT within its intended, real-world setting. No study personnel were present for this testing. Participants used the same suite of software programs as in Phase 1 to monitor surgical patients in real time and address the AlertWatch Tower Mode alerts. They did not interact with clinicians in the ORs. A secure server captured and stored a log of all alerts and participant responses. Testing sessions were of one business day duration. Based on the anticipated staffing model for the intervention, attending anesthesiologists participated one day at a time on days in which they were not assigned to the surgical ORs. Resident anesthesiologists participated for 10 consecutive business days as part of a formal 2-week rotation during their final year of clinical training. All participants completed the SUS [44], CSUQ [45], and NASA-TLX [46]. Attending anesthesiologists completed the questionnaires every day that they were in the ACT. To minimize the degree to which resident anesthesiologists were biased by their previous questionnaire responses, they only completed the surveys 3 times over the course of their rotations rather than on a daily basis.

#### Clinician Interviews

Phase 3 involved semistructured interviews with clinicians who were potential recipients of ACT feedback. After an initial orientation to the ACT, participants were prompted to provide their initial impressions of the intervention. Subsequently, a research team member presented six examples of scenarios for which participants were instructed to imagine themselves as the actual recipient of ACT feedback in each scenario. Five of the scenarios involved clinical alerts; the final scenario included a billing alert. After participants verbalized their initial reaction, the team member used a short series of open-ended questions to obtain input about the usefulness of each individual alert as well as the preferred delivery mode (eg, text, page, phone call). A debriefing session used open-ended questions to ascertain participants’ final impressions of the ACT and their feedback on specific components of the ACT intervention. All interview sessions were audio recorded and professionally transcribed, with transcriptions verified against the original audio recordings.

### Data Analysis

#### Quantitative Analysis

Participant characteristics from all three phases were analyzed using descriptive statistics. In phase 1, the frequency with which participants experienced a critical usability issue that required an intervention to continue the session was determined. Performance measures for the two ACT clinician usability testing phases were summarized with mean and standard deviation. These measures included time to task completion (phase 1) and quantity and rate of task completion (phases 1 and 2) [36,47]. For phase 2 testing, performance measures were analyzed across iterations of the software platform. Subjective measures of usability and workload in phases 1 and 2 were summarized with mean scores for the SUS and standard deviation for the CSUQ and NASA-TLX. Results from these surveys were compared between attending and resident physicians and between initial and repeat testing sessions. As a measure of participant satisfaction with the built-in software alerts, the percentage of alerts in phase 1 and phase 2 that were classified as significant or potentially significant was determined. Statistics were calculated using SPSS Statistics for Macintosh (IBM Corp).

#### Qualitative Analysis

Research team members used NVivo 12 software (QSR International Pty Ltd) to perform a qualitative content analyses of the ACT clinician think-aloud sessions and the OR clinician semistructured interviews. They analyzed transcripts in order to identify themes regarding the usability of the ACT (phase 1 ACT clinician sessions) and barriers and facilitators to its implementation (phase 3 OR clinician interviews). First, one researcher (TM-T) generated separate codebooks for each set of qualitative data (see Multimedia Appendix 2), and additional team members (AC, MB, and MP) helped refine them. Two researchers (TM-T and AC) double-coded transcripts until a kappa of at least .75 and percentage agreement of at least 97% had been obtained. Four transcripts from phase 1 and three transcripts from phase 3 were double coded. Subsequently these researchers coded the remaining transcripts independently. If consensus could not be reached during coding, a third team member (MP) reviewed the categorization.

Qualitative content analysis for phase 1 usability testing began with segmentation of the verbal data and ended with interpretation [48]. The final coding scheme (Multimedia Appendix 2) contained seven previously employed think-aloud content domains [49-54]. Analyses of the semistructured debriefing sessions focused on 2 of the 7 content domains (user experience, redesign proposal). In the final round of analyses, usability problems and redesign proposals were extracted from the transcripts [52,54]. Individual problems were placed into 1 of 4 categories (navigation, content, functionality, and layout; Multimedia Appendix 2). Redesign proposals were similarly grouped and compared with the themes from the usability problem set.

The analysis for phase 3 consisted of a qualitative usability analysis and thematic analysis. During this phase, one team member (TM-T) reviewed transcripts to explore participants’ evaluation of the usefulness of the ACT in each individual clinical scenario in addition to the communication preferences of the participants. We based the thematic analysis on the theoretical domains framework that has demonstrated utility in examining constructs related to behavior change in a variety of health care settings [29,55-58]. After coding was complete, the researcher (TM-T) reviewed all utterances and created belief statements that captured meaningful themes within each domain [59]. These belief statements were summarized across participants and reviewed by two other team members (AC and MP).

## Results

### Participant Characteristics

Table 2 shows the characteristics of the participants at each stage of the testing process. A total of 32 clinicians participated over the course of all phases of testing. Eight attending and seven resident physician anesthesiologists participated in phase 1 testing; six attending and eight resident physician anesthesiologists participated in phase 2. Four attending physicians and six CRNAs participated in the semistructured OR clinician interviews. Resident physicians were also recruited for these interviews; however, due to challenges related to scheduling, no resident physicians were able to participate in the sessions.

### Quantitative Data

Participants evaluated an average of 7.25 patients and addressed an average of 11.5 alerts per session during the phase 1 think-aloud usability sessions. During the phase 2 real-world testing, participants evaluated an average of 54.9 unique patients per day and addressed an average of 176 alerts across all platforms. Of the alerts addressed each day in phase 2, on average 40.3% (50/124) of them were repeat alerts—that is, one specific alert that triggered repeatedly for a single patient during a single operation.

The mean overall score for the SUS across all phase 1 and phase 2 sessions was 66.3, below the threshold of 70 that indicates a sufficient level of satisfaction [60]. The score tended to be higher during testing sessions that were repeat sessions versus the initial session (70 [SD 15] vs 62.6 [SD 16]) and for resident physicians versus attending physicians (70.3 [SD 14.9] vs 62.9 [SD 16.3]). Workload as measured by the NASA-TLX followed a similar pattern; lower workload was measured during repeat testing sessions (46.5 [SD 15.3] vs 53.2 [SD 18.3]) and among resident physicians versus attending physicians (50.1 [SD 12.9] vs 58.5 [SD 19.7]). No significant differences were found in CSUQ total or subscale scores between any testing conditions or participant roles. With regard to participant satisfaction with the specific software-generated alerts, participants determined that only 27.05% (680/2513) of the alerts generated by the first iteration of the platform were actually clinically significant or potentially significant. In subsequent iterations of the platform, this proportion of clinically useful alerts improved to more than half to three-quarters of all software-generated alerts (range 56.00% [933/1666] to 73.05% [1640/2245]).

**Table 2:** Characteristics of participant groups.

Characteristics			Attending anesthesiologists (n=16)	Resident anesthesiologists (n=10)		Certified registered nurse anesthetists (n=6)
**Participants at each phase^a^ (n)**						
	Phase 1 ACT^b^ clinician think-aloud sessions	8		7	N/A^c^	
	Phase 2 ACT clinician real-world testing	6		8	N/A	
	Phase 3 OR^d^ clinician interviews	4		0	6	
Years at institute, average (range)			6.6 (0.75-21)	11.8 (3-22)		3.9 (3-4)
**Sex, n (%)**						
	Male	10 (63)		5 (50)	3 (50)	
	Female	6 (38)		5 (50)	3 (50)	
**Baseline AlertWatch use, n (%)**						
	Almost always	1 (6)		2 (25)	1 (17)	
	Sometimes	6 (38)		3 (33)	2 (33)	
	Rarely or never	8 (50)		2 (25)	3 (50)	

^a^There was an overlap of nine participants between phase 1 and phase 2 and two participants between phase 2 and phase 3. No participants overlapped between phase 1 and phase 3.

^b^ACT: Anesthesiology Control Tower.

^c^N/A: not applicable.

^d^OR: operating room.

### Qualitative Data

#### Usability Problems

A total of 155 usability problems were identified in the phase 1 transcripts (Table 3), the majority of which were related to functionality (57/155, 36.8%) and content (53/155, 34.2%), followed by navigation (29/155, 18.7%) and layout (16/155, 10.3%). Three participants experienced a critical usability issue that required an intervention on the part of the research team member in order to continue in the session. Two of these participants were unable to locate the help documents that provided instructions on how to access the software programs, and the third participant loaded the wrong software platform. All three participants were able to continue in their sessions once the researcher pointed out the location of the help documents on the computer desktop.

The remaining usability problems were associated with delays in task performance or had minor effects on the testing session. Many users had difficulty both in understanding the meaning or relevance of the software-generated alerts (8/15, 53%) and determining the alert severity or priority (7/15, 46%). Some (7/15, 46%) noted being distracted by what they viewed as irrelevant or repeated alerts, and a few (3/15, 20%) reported being overwhelmed by the sheer number of alerts that they faced. Most clinicians reported limitations in their ability to address alerts and monitor patients due to poor interoperability and lack of integration of the different software programs (6/15, 40%) as well as slow response times for the software programs themselves (7/15, 46%). The fewest usability problems were associated with layout and were focused on participants’ inability to move applications to the preferred of three monitors (3/15, 20%) or resize the program windows (2/15, 20%).

#### Redesign Proposals

Redesign proposals or suggestions for improvements were described by 12 of the 15 participants, with a total of 51 proposals reported from all participants. The proposals were often associated with the usability problems that participants encountered. Examples of these redesign proposals are provided in Multimedia Appendix 3. The majority of proposals were related to content (32/51, 63%) and included suggestions for improving alert relevance (13/51, 25%) and alert prioritization (9/51, 18%). Seven iterations of the AlertWatch Tower Mode platform (described in Multimedia Appendix 4) were tested during the trial and reflected the proposals generated by participants. The alterations included refinements in the visual display and presentation of information and changes in alert content and prioritization (shown in Multimedia Appendix 3).

#### Operating Room Clinician Perspective on Usability

In phase 3, the five clinical scenarios presented to participants were considered to be useful or potentially useful by all (10/10, 100%, for two of the scenarios) or almost all (8-9/10, 80%-90%, for the remaining scenarios) of the participants. Clinicians often had suggestions for how the usefulness of alerts could be improved, and many offered additional scenarios in which they would be satisfied with the usefulness of the ACT. Participants generally agreed that the preferred method of contact would depend on the clinical scenario; minor alerts could be sent through text, page, or even through the creation of a novel computer pop-up, whereas major alerts could be delivered by phone. The general consensus was that in order for a method of communication to be useful, it could not increase the provider’s workload, distract from their current tasks, or add to their alarm fatigue.

**Table 3:** Usability problems identified in the Anesthesiology Control Tower clinician think-aloud and debriefing sessions.

Category^a^ and theme		Number reporting	Example quotation
**Navigation**			
	Trouble finding link or information	5	“Okay, so I have already forgotten what the heck I’m supposed to do to respond. I need to get that thing where I can click on ‘responses’ and I don’t remember where it is.” [Participant 2127, attending physician]
	Unable to determine which link to use	2	“I don’t know the difference between [two log-in options]. I don’t know which one to do.” [Participant 2108, attending physician]
	Selected incorrect patient or operating room	2	“So here I was accidentally using the last patient we had, looking at that patient, before I realized that I was not on the correct patient.” [Participant 2114, attending physician]
	Any navigation problem	9	—^b^
**Content**			
	Alert meaning or relevance unclear	8	“I'm unclear as to what infusions 4.0 means—whether that means 4 different types of infusions? ...I'm not sure what this means.” [Participant 2105, resident physician]
	Difficulty prioritizing alerts	7	“Which is worse, black or red? I’m guessing red...that wasn’t spelled out to me, but I’m going to say yes.” [Participant 2127, attending physician]
	Information not available	6	“We are basically looking at a blank sheet with blood pressures randomly listed. I am unable to make any sort of reasonable clinical judgment at this point.” [Participant 2114, attending physician]
	Unable to identify correct patient or operating room	4	“What OR^c^ is this again? I forgot what OR it is.” [Participant 2112, resident physician]
	Any dialogue problem	11	—
**Functionality**			
	Poor software response times	7	“Waiting for [anesthesia information management system] to log in. Still waiting.” [Participant 2127, attending physician]
	Limited interoperability of software programs	7	“I’m a little frustrated because right now it seems kind of a hassle to access all these programs to make a simple decision.” [Participant 2110, attending physician]
	Inability to manipulate location of software programs on screen	3	“[The anesthesia information management system] won't let me move it to another screen. Looks like that is stuck on my middle screen, where [the EHR] I was able to move from monitor to monitor.” [Participant 2106, attending physician]
	Difficulty logging in to programs	5	“For some reason it does not allow me to log in or use [hospital] access.” [Participant 2101, attending physician]
	Any functionality problem	13	—
**Layout**			
	Text not visible	4	“I’ll have to spend a minute here trying to cover my cursor over to read the full case...chest wall reconstruction. It’s sort of hard because it keeps going away.” [Participant 2114, attending physician]
	AlertWatch does not fit	2	“I first noticed AlertWatch is off the screen a little bit, trying to see if I can make it fit better—it doesn’t really fit.” [Participant 2103, attending physician]
	Physical layout (monitors)	3	“How do I get the big board on the big screen? On the right? By convention it should be on the left.” [Participant 2103, attending physician]
	Any layout problem	9	—

^a^Adapted from Zhao et al [54].

^b^Not applicable.

^c^OR: operating room.

#### Barriers to Implementation

The interviews with OR clinicians in phase 3 generated 33 summary belief statements (Multimedia Appendix 5). Of these belief statements, 20 addressed potential barriers to the ACT implementation. Several participants questioned the necessity of the ACT and whether there were other ways to support good clinical care that would not require a “control tower.” Many could imagine themselves to be frustrated or annoyed with a poorly executed intervention (emotion). They reported that the usefulness of even well-designed and accurate alerts could be drastically limited if the alert were poorly timed and distracted the clinician from meaningful patient care tasks (beliefs about consequences).

Many participants viewed the ACT intervention as being in actual or potential conflict with their professional autonomy as clinicians. Several participants feared the downstream impact of the ACT on provider satisfaction and even the department’s ability to recruit and retain talented personnel (social professional role and identity, social influences, beliefs about consequences). Some also expressed apprehension regarding the very concept of remote monitoring (social professional role and identity) and imagined that their colleagues would feel similarly (social influences). A few participants questioned how the ACT would integrate into the legal structure for the provision of anesthesia and whether it would disrupt existing relationships between members of the anesthesia care team (beliefs about consequences).

Attending physicians and CRNAs voiced concern that the ACT support would be redundant, and some doubted the ability of the ACT to provide useful information of which the provider was not already aware. Several clinicians also imagined that the ACT clinicians may not be able to understand a patient’s comorbidities and anesthetic needs as well the primary team did themselves (memory, attention, decision processes). In this setting, they stated that the ACT could worsen their workload if they had to take additional time to provide the missing information that would have allowed the ACT to better understand the patient’s situation (beliefs about consequences). Additionally, participants worried that current limitations or flaws in monitoring and software systems could lead to the generation of false alarms or prevent clinicians from being able to act meaningfully on the ACT support (environmental context and resources). Participants identified flaws in the communication modalities currently available at the hospital which they envisioned leading to impairments in the ACT’s ability to deliver timely and useful information (environmental context and resources).

#### Facilitators of Implementation

Despite potential barriers to implementation, all of the clinicians were able to identify several specific instances in which they could see benefit from the ACT intervention. In general, participants agreed that a timely alerting system that did not increase their workload or interrupt patient care could be useful. Attending physicians stated that the ACT could be useful for them during times when they were covering multiple busy rooms, either notifying them of acute major events or of smaller, but still relevant, alerts in stable cases (social professional role and identity). The ACT was also thought to be particularly helpful for newly employed or inexperienced clinicians (social professional role and identity, beliefs about consequences). In true crises, participants stated that the ACT could be most useful in helping the OR clinician to obtain additional hands-on assistance or by reviewing electronic records for critically relevant information that the OR team could be missing in the midst of a dynamic clinical situation (memory, attention, decision processes).

Almost all of the clinicians agreed that the clinical practices described in the example scenarios were consistent with good anesthesia practice (knowledge, nature of behavior). Most reported that the concept of the ACT intervention was simple to understand (knowledge). Clinicians identified patient safety as a focus of their identity as an anesthesia provider and stated that any interventions that enhanced this would be welcome (social professional role and identity, beliefs about consequences). In contrast to the clinicians who were apprehensive regarding the concept of remote monitoring, some participants clearly expressed willingness to incorporate the intervention into practice at the hospital. One provider compared the ACT to telemedicine in the intensive care unit, reporting this as a positive factor in having another clinician watching out for them and the patient (optimism).

## Discussion

### Principal Findings

In this paper, we have described a thorough and iterative evaluation of a novel telemedicine-based intervention for the OR from the perspective of key groups of end users. Our findings related to usability problems and barriers to implementation are consistent with prior studies investigating the incorporation of novel information technologies into clinical practice, and they allowed us to refine aspects of the intervention prior to the initiation of a pragmatic randomized controlled trial. Previous studies have demonstrated the necessity of comprehensive usability testing before the implementation of health information technologies into routine practice. As one group of authors noted, “it would be unthinkable that the airline industry would have its first trial of an airplane’s flight capabilities with real passengers” [61]. The usability testing that we performed in this study allowed us to “pilot” the ACT prior to its implementation, enabling us to identify and mitigate limitations arising from the technical aspects of the intervention.

Participants identified a number of surface-level usability problems during phase 1 usability think-aloud sessions [62]. Usability problems were often related to visual displays and software interfaces, limited availability of information, and poor interoperability of software programs, consistent with prior work introducing novel technologies into clinical practice [63,64]. The phase 2 real-world usability testing provided complementary insight into usability and workflow concerns in a realistic environment [65,66]. Participants identified similar concerns to those discussed during the think-aloud sessions, such as difficulty prioritizing alerts and receiving repetitive alerts for a single patient. The iterative changes that were made to the ACT intervention (Multimedia Appendix 3) improved the interface and its interactive features [23]. The decrease in the number of insignificant alerts reduced participants’ cognitive load and alarm fatigue [23], allowing them to focus more on addressing clinically meaningful situations.

Although many usability problems were addressed over the course of testing, the research team was unable to improve the communication between the individual software programs used by the participants. In order to understand the context of an alert generated by the AlertWatch Tower Mode software, participants almost always required supplementary information from the hospital EHR and the anesthesia information management system. This process required clinicians to manually load individual patients into each of these different software programs. This lack of a uniform system was a known limitation of the EHRs at our institution at the time this study was performed. Recently, however, our institution recently transitioned from multiple EHRs to a single, combined platform, and we anticipate that the change will address this limitation.

Semistructured interviews with potential recipients of the ACT support discovered a range of beliefs related to facilitators and barriers to implementation of the ACT similar to findings from previous studies [30,56,58,59]. Our participants identified a number of potential adverse consequences to the introduction of a new technology and set of processes into routine care [67], including concerns regarding increased work for clinicians, unfavorable workflow issues, untoward changes in communication patterns and practices, negative emotions, and unexpected changes in the power structure. Some of these barriers indicate reactance on the part of the clinicians; that is, they experienced negative reactions as a result of threats to their autonomy and freedom of choice [68]. In response to some of the barriers involving professional autonomy and the social roles and identities of the study participants, the final ACT intervention was modified from a physician-only staffing model to one that also incorporated the clinical expertise of certified and student nurse anesthetists. This staffing model reflects the current structure at our institution in which attending physicians, nurse anesthetists, and resident physicians play important and complementary roles in providing care to surgical patients.

Despite the number of barriers revealed during testing, participants identified several facilitators to implementation such as a cultural commitment to patient safety. Participants expressed a willingness to engage with the ACT in order to improve its applicability and usefulness in helping clinicians adhere to high standards for patient care. Suggestions regarding the timing and appropriateness of specific alerts were consistent with the research team’s initial design for the ACT and were incorporated into the final intervention.

### Limitations

Results of the study should be interpreted within the context of several study limitations. Participants worked in one academic center that may not be representative of all health care settings. Study participants in the different phases were not representative of the final staffing model for the ACT. Specifically, the initial, physician-only staffing model for the ACT led to the exclusion of CRNAs from phase 1 and phase 2 usability testing. Additionally, resident physician anesthesiologists did not participate in phase 3 due to scheduling conflicts with their daily assignments. The lack of input from CRNAs in phases 1 and 2 may have prevented us from identifying all relevant usability problems related to the ACT intervention. However, due to similar backgrounds and experiences with the hospital system’s EHRs between the physician anesthesiologists and CRNAs, we anticipate that testing with the two groups might have identified similar usability problem themes. We did obtain feedback from CRNAs during the semistructured interviews regarding the usability of the ACT from the OR clinician perspective. Across all testing phases, selection bias may have resulted in participants who felt most strongly about the ACT intervention being more or less inclined to participate. Finally, no patient-related outcomes were included in this study [19]; however, an ongoing randomized controlled proof-of-principle trial at our institution is evaluating metrics of care quality and tracking patient outcomes.

### Conclusions

This mixed-methods study explored concerns about incorporating an innovative telemedicine-based clinical support system into routine clinical practice. Consistent with recommendations for assessing complex health interventions prior to their implementation, this study conducted usability testing and an analysis of barriers to and facilitators of implementation based on a theoretical framework [31,32]. By assessing not only usability but also acceptability and relevance [22,32] for two groups of end users, we maximized the potential of the ACT intervention to provide clinicians with the right support, in the right format, at the right time in the care continuum [69], thereby enhancing the ability of the ACT to meaningfully impact patient care.

## Appendix

Multimedia Appendix 1 Anesthesiology Control Tower description.

Multimedia Appendix 2 Phases 1 and 3 study session codebooks.

Multimedia Appendix 3 Summary of changes to the AlertWatch Tower Mode platform.

Multimedia Appendix 4 Redesign proposals offered by clinicians in phase 1.

Multimedia Appendix 5 Summary of relevant belief statements and sample quotes from phase 3 clinicians.

## Abbreviations

ACT: Anesthesiology Control Tower
ACTFAST: Anesthesiology Control Tower—Feedback Alerts to Support Translation
CRNA: certified registered nurse anesthetist
CSUQ: Computer System Usability Questionnaire
EHR: electronic health record
NASA-TLX: NASA Task Load Index
OR: operating room
SUS: System Usability Scale
