# Supplementary material for: Anesthesiology Control Tower—Feasibility Assessment to Support Translation (ACTFAST): Mixed-Methods Study of a Novel Telemedicine-Based Support System for the Operating Room
Source: JMIR Hum Factors. 2019 Apr 23;6(2):e12155. doi: 10.2196/12155 (PMC6658281; doi:10.2196/12155)
Supplement: Multimedia Appendix 1 [file humanfactors_v6i2e12155_app1.pdf]

Multimedia Appendix 1: Anesthesiology Control Tower description

We previously included a description of the Anesthesiology Control Tower in our publication of our study protocol[1]. The description is repeated here for ease of access to the information.

Study context

The ACTFAST1 study is one component of a larger effort to implement the Anesthesiology Control Tower (ACT) into routine practice. It was performed prior to the initiation of a proof-of-principle pragmatic randomized controlled trial that is currently ongoing (NCT02830126 [3]) and aims to demonstrate the utility of the ACT in improving adherence to clinically relevant practice guidelines, and to evaluate the effect of the ACT on surrogate measures of patient outcome. Each of our institution's adult operating rooms (ORs) are randomized on a daily basis to either a control group or to an experimental group in which anesthesia providers receive the support of the ACT. We are also conducting a concurrent study [2] that is working to develop, refine, and validate forecasting algorithms to predict negative patient trajectories; such algorithms will serve to help inform future ACT interventions.

ACT technology

The Anesthesiology Control Tower (ACT) was originally staffed by attending and resident anesthesiologists; the staffing model was expanded to include certified registered nurse anesthetists (CRNAs) and student registered nurse anesthetists. The ACT was created by taking advantage of a broad range of information technologies that are available at our institute. These include the hospital's electronic health records (EHRs) including the anesthesia information management system (Metavision by iMDsoft®, Needham, MA), treatment guidelines and protocols for care, and the AlertWatch® (Ann Arbor, MI [4]) software, all of which are accessible remotely on any secure device with Internet connection. In the ACT, trained clinicians

(physician anesthesiologists and certified registered nurse anesthetists) use these technologies to monitor, in real time, ongoing surgical cases at our institute.

Monitoring in the ACT is guided by a modified "Tower Mode" version of the FDA-cleared AlertWatch® platform (Kl3O4Ol). AlertWatch® is a decision support software device that integrates information from patient monitors and EHRs, analyzes the data to determine the current patient state, and displays this information on a patient display dashboard (Figure 1); the "Tower Mode" was created for the purposes of the ACT and modified based on the results of the ACTFAST1 study.

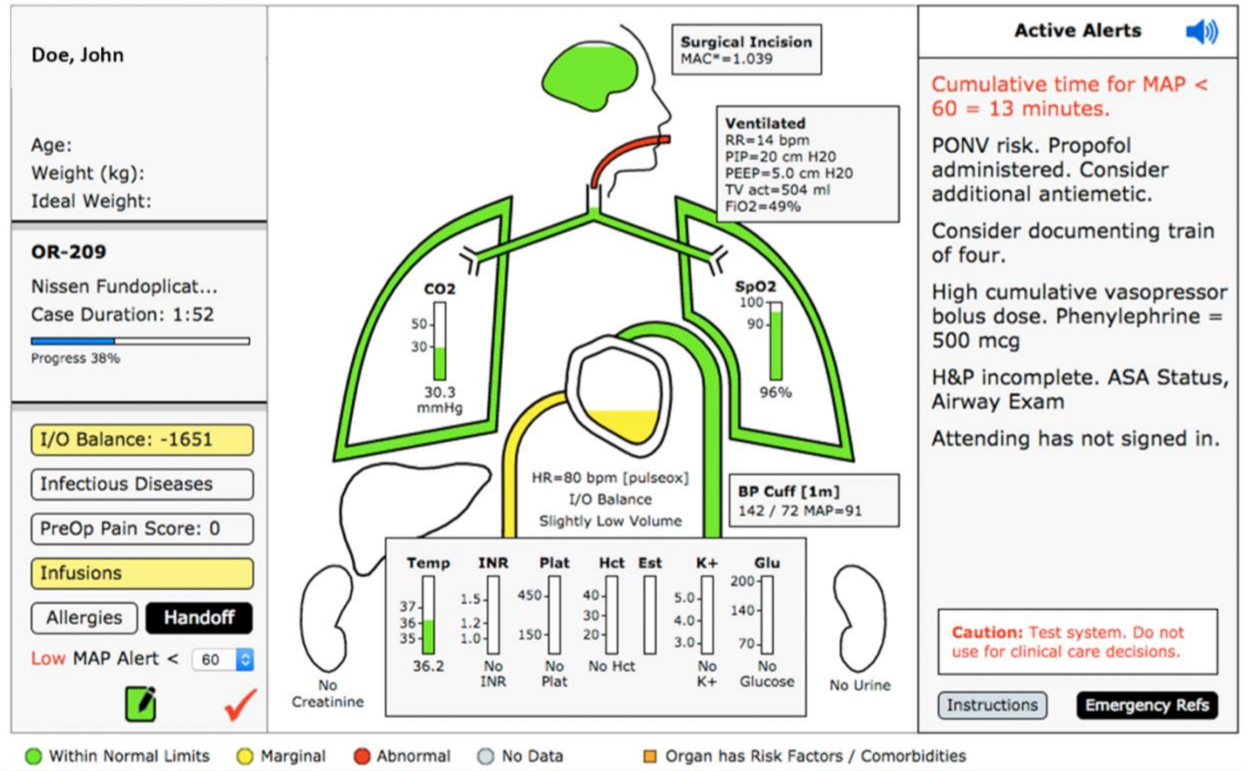

Figure 1. AlertWatch® Tower Mode patient display. The left side of the display shows patient characteristics and surgical case information. Organ systems are depicted and labeled with relevant physiologic variables and values. Information about the current function of an organ system is indicated by the fill of the organ; colors indicate normal (green), marginal (yellow) or abnormal function (red). Information regarding the actual patient's comorbidities can be accessed by selecting the organ system or laboratory study of interest. Text alerts are presented on the right hand side of the screen. In this figure the red checkmark

at the bottom of the left panel indicates that there is an active, actionable alert for the ACT clinicians to address. Clicking on the checkmark opens the Case Review dialogue that is used by ACT clinicians to document their assessment and response to the alert (Figure 3).

When calculated variables or incoming data suggest the potential for an adverse outcome, rules within AlertWatch® trigger alerts or reminders to appear in the operating room (OR) of interest. These alerts are indicated by several different symbols on a main census view (Figure 2); the specific symbol shown is dependent on the nature of the alert.

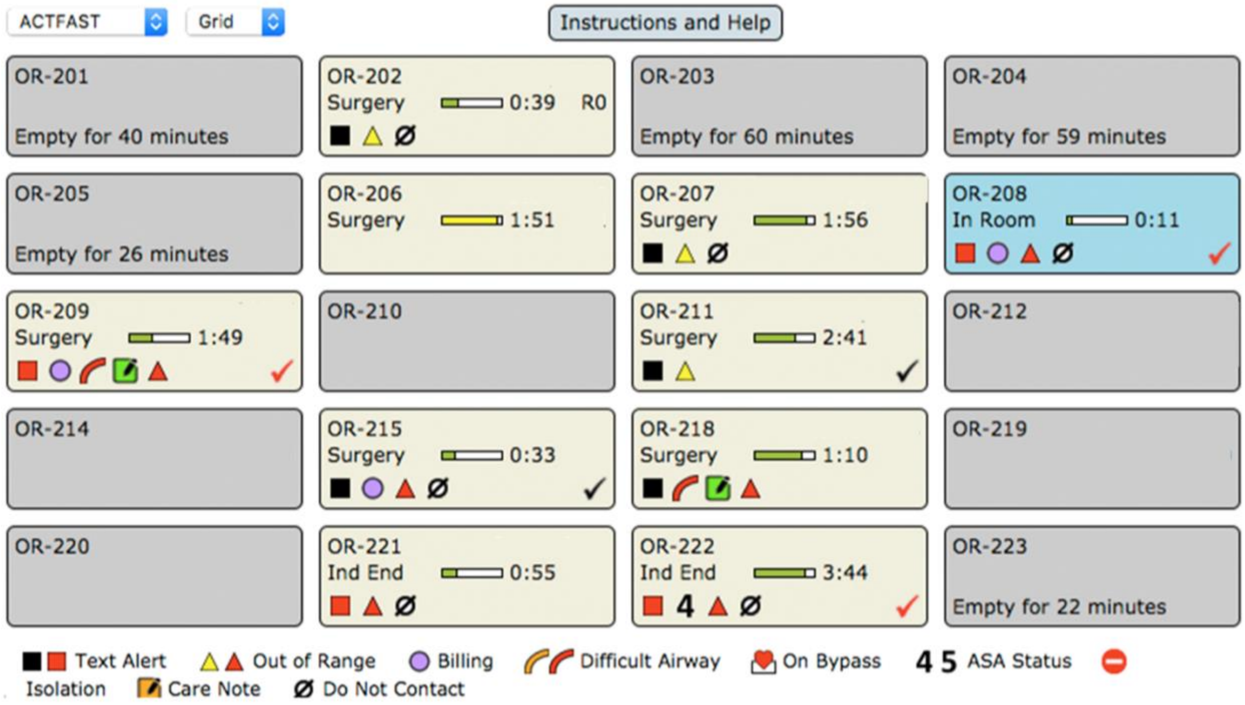

Figure 2. AlertWatch® Tower Mode census view. From this view, clinicians in the ACT can obtain a brief overview of all the patients in the ORs. Alerts and abnormal physiologic and laboratory parameters are represented by squares and triangles, respectively; checkmarks indicate specific, "actionable" alerts that must be addressed by the ACT (Figure 3). These groups of alerts are unique to the AW Tower Mode and were refined based on the results of the present study.

Alerts that are considered to be actionable generate a checkmark both on the patient display (Figure 1) and on the census view (Figure 2). Clinicians in the ACT are instructed to respond to

these alerts, and they do so by obtaining additional information from the EHRs and the anesthesia information management system in order assess the relevance and importance of the incoming information. If the alarms are deemed to be clinically relevant and significant, the ACT clinicians send supplementary notifications to the involved clinicians in the OR. Clinicians in the OR are not forced to follow any recommendations sent by the ACT, and the final decision regarding the applicability of the alert is made by the primary team providing care for the patient. ACT clinicians document their assessment of these alerts and their interaction with the OR clinician in a Case Dialogue window in the AlertWatch® Tower Mode (Figure 3).

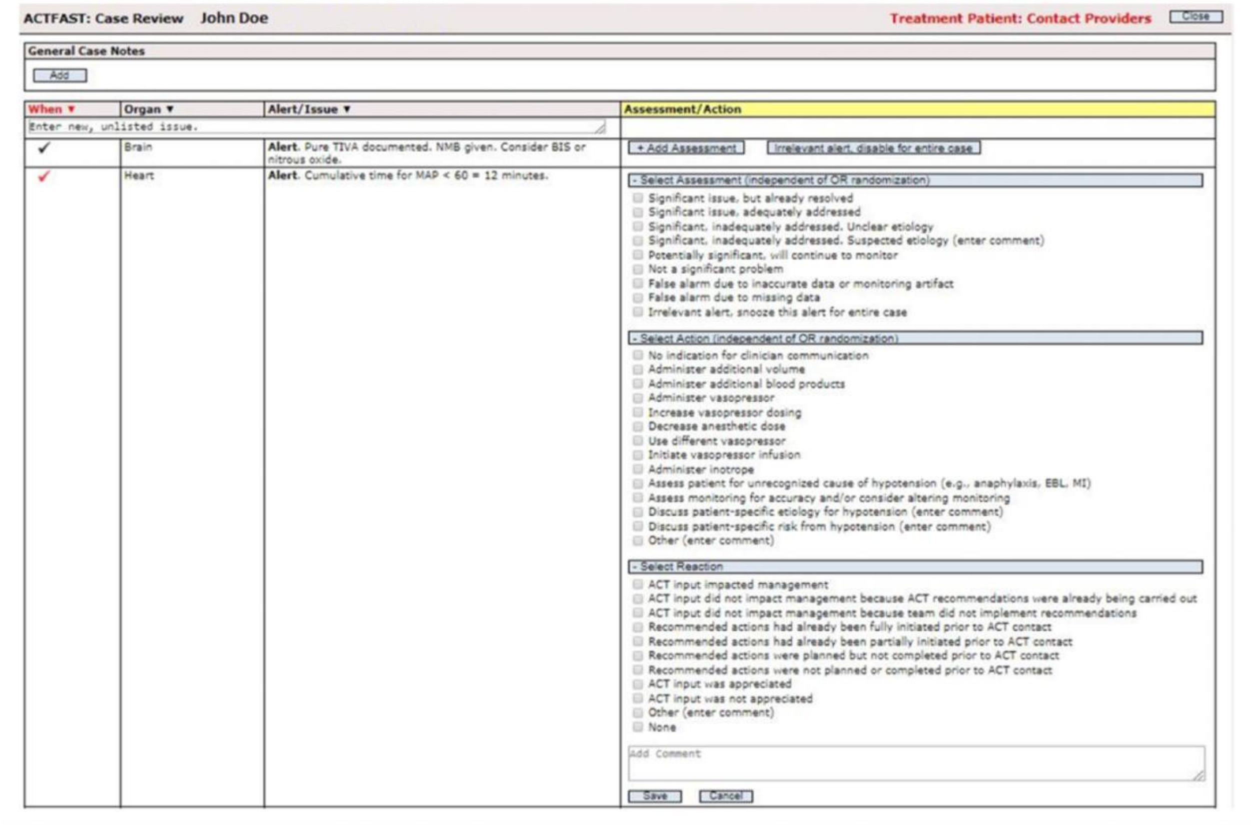

Figure 3. AlertWatch® Case Review. This popup window allows clinicians in the ACT to document their assessment of alerts and what actions they would recommend. This is a feature of AlertWatch that is unique to the ACT Tower Mode platform. ACT clinicians assess and address an alert by documenting their assessment of the significance of the alert and by documenting what action they would recommend taking, if any.

All of the actionable checkmark alerts that are included in the AlertWatch® Tower Mode platform were chosen because they follow proven best intra-operative management practice and are in line with national metrics for quality and safety [5-7]. Specific alerts are based on large prospective studies that have established outcomes benefit with a specific clinical management paradigm (e.g., timing of antibiotics, blood transfusion triggers, avoidance of hypothermia). Importantly, the programmed alerts that are available in the Tower Mode platform are dynamic and can be re-programmed and adapted.

Integration into standard practice patterns

The model for anesthesia staffing at our institute is one in which resident physicians or CRNAs provide direct clinical care to surgical patients in individual ORs, adhering to standards established by the American Society of Anesthesiologists. Attending anesthesiologists provide supervision or direction for as many as three to four operating rooms simultaneously (Figure 4). Anesthesia clinicians monitor the status of an individual patient by filtering and analyzing a wide range of incoming data from numerous sources such as the physical exam of the patient, standardized monitors and alarms, the anesthesia information management system and other EHRs, and interactions with the surgical team. These clinicians are often inundated with alarms and alerts. Practitioners may see alarms as frequently as every 3 minutes—even more frequently during induction of anesthesia and emergence from anesthesia—and while the majority of alarms might appear to be clinically irrelevant, a small percentage do require immediate intervention. In this setting, alerts or alarms generated by the ACT could become simply another level of "noise" in an environment with a known predisposition for information overload. There are two key features of the ACT that aim to prevent this occurrence. The first is the presence of the modified AlertWatch Tower Mode alerting system platform that, in the future, will be aided by advanced

machine-learning adaptive algorithms [2]. The second is the presence of skilled clinicians who are able to filter and prioritize information in order to improve the quality of alerts and to limit false alarms to the clinicians in the OR. This conceptualization of the ACT allows it to decrease the burden from false and intrusive alarms in the OR, and instead provide an empowering and unobtrusive IT-based decision support solution to clinicians.

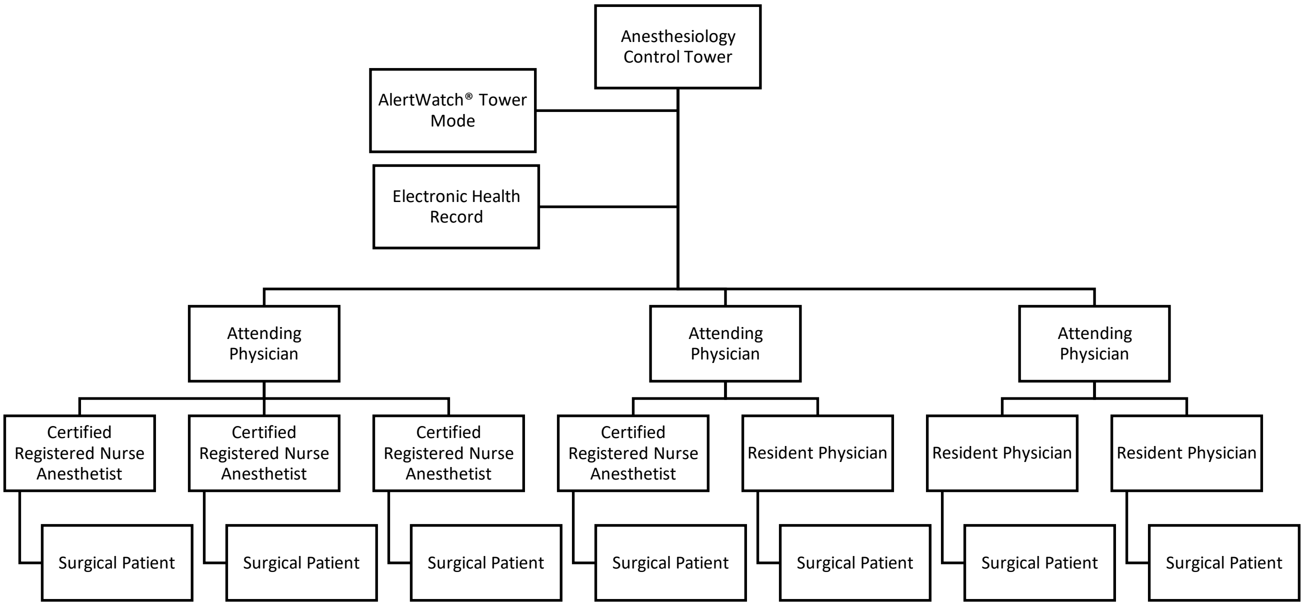

Figure 4. Diagram of ACT integration into OR process. The common clinical model for anesthesia services at our institute consists of resident and fellow physicians and CRNAs who are supervised or directed by an attending physician. Attending physicians cover up to three rooms simultaneously. In-room providers use a range of data sources to monitor the current status of their patient. In the ACT, the Census View in AlertWatch® (Figure 1) allows ACT clinicians to track clinical notifications and alerts that are generated in real time. Clinicians obtain additional information from the EHR and the Patient Display (Figure 2) to assess the relevance and importance of the incoming information. When indicated, physicians in the ACT communicate decision support to anesthesiology clinicians in the OR via pagers and dedicated telephones.

Physical structure

The ACT is physically housed within the hospital complex, remote from the physical operating rooms. This room includes 3 computers; two with three monitors and one with two monitors. A large flat-screen TV displays the AlertWatch® census view. The presence of additional monitors allows multiple AlertWatch® windows to be visible, in addition to providing space for additional software programs. All software (i.e. our electronic health record, our anesthesia information management system, and AlertWatch®) is accessible on these computers either through desktop icons or links through our department's webpage. A introductory guide to the ACT and an AlertWatch® Tower Mode help guide are provided in a physical format in addition to electronic copies. Two landline telephones are available for use in contacting clinicians in the ORs.

References

1. Murray-Torres TM, Wallace F, Bollini M, Avidan MS, Politi MC. Anesthesiology Control Tower: Feasibility Assessment to Support Translation (ACT-FAST)—a feasibility study protocol. Pilot and Feasibility Studies. 2018;4(1):38. PubMed PMID: 29416871.

2. Fritz BA, Chen Y, Murray-Torres TM, Gregory S, Ben Abdallah A, Kronzer A, McKinnon SL, Budelier T, Helsten DL, Wildes TS, Sharma A, Avidan MS. Using machine learning techniques to develop forecasting algorithms for postoperative complications: protocol for a retrospective study. BMJ open. 2018;8. PubMed PMID: 29643160.

3. Gregory S, Murray-Torres TM, Fritz BA, Abdallah AB, Helsten DL, Wildes TS, Sharma A, Avidan MS, Group AS. Study protocol for the Anesthesiology Control Tower—Feedback Alerts to Supplement Treatments (ACTFAST-3) trial: a pilot randomized controlled trial in intraoperative telemedicine. F1000Research. 2018;7. PubMed PMID: 30026931.

4. Tremper KK, Mace JJ, Gombert JM, Tremper TT, Adams JF, Bagian JP. Design of a Novel Multifunction Decision Support Display for Anesthesia Care: AlertWatch® OR. BMC anesthesiology. 2018;18(1):16. PubMed PMID: 29402220.

6 Surgical Care Improvement Project Core Measure Set: Centers for Medicare & Medicaid Services and the Joint Commission; 2014 [3/20/2015]. Available from: http://www.jointcommission.org/assets/1/6/SCIP-Measures-012014.pdf.

6. Specifications Manual for National Hospital Inpatient Quality Measures 2015 [3/20/2015]. Available from: http://www.jointcommission.org/specifications_manual_for_national_hospital_inpatient_quality_measures.aspx.

7. ASA. Statement on the surgical care improvement project. American Society of Anesthesiology Performance and Outcomes Measurement Committee; 2010.
8. de Man FR, Greuters S, Boer C, Veerman DP, Loer SA. Intra-operative monitoring--many alarms with minor impact. Anaesthesia. 2013;68(8):804-10. PubMed PMID: 23745968.
