# Supplementary material for: Anesthesiology Control Tower—Feasibility Assessment to Support Translation (ACTFAST): Mixed-Methods Study of a Novel Telemedicine-Based Support System for the Operating Room
Source: JMIR Hum Factors. 2019 Apr 23;6(2):e12155. doi: 10.2196/12155 (PMC6658281; doi:10.2196/12155)
Supplement: Multimedia Appendix 2 [file humanfactors_v6i2e12155_app2.pdf]

Multimedia Appendix 2. Phase 1 and Phase 3 coding schemes

Table 1. Phase 1 ACT clinician usability testing: codebook for categorizing utterances.

Topic	Definitions	Decision rule	Examples
**ACTION DESCRIPTION** [1-6]	Descriptions of what participants are doing, trying to do, or did.	Code<br>Verbalizations of what actions participants were taking or were trying to take, **without an indication of *why* they did** it or *what they experienced* while doing it.<br>Includes verbatim *reading out loud of words, phrases, and sentences from the screen*.<br>Includes descriptions of what links or programs are being selected by the participant.<br>Do not code<br>Verbalizations regarding the visual display that are not strictly reading from screen should be coded at Observation.<br>If participant is describing what the software is doing (i.e. loading or freezing) and not what *they themselves* are doing, code under Observation.<br>If the participant states that they are opening a link or a program *in order to do something other than just opening it*, code at explanation.<br>If a participant is having difficulty performing a specific action, code at user experience.	*"So now I'm opening Metavision and I'm clicking on the Metavision icon, and I'm hitting "open". I don't want to store my password."*<br>*"Lastly, it says infusion 4.0."*<br>*"I'm going to open a new Chrome window here and open up AlertWatch."*<br>*"Clicking on Metavision to open it."*<br>*"So now I'm opening Metavision and I'm clicking on the Metavision icon"*<br>*"I'm clicking on "Main Chart", and I'm clicking on every 15 minute increments"*
**OBSERVATION** [1, 2]	Verbalizations making observations of software system platforms, including their features and layout, and the information they display.	Code<br>Verbalizations that describe the software platforms and their visual interfaces without providing information on what the participant was doing, why they were doing it, or what they were experiencing.<br>Comments on information presented on the screen for a software program, including what is present or how it is arranged.	*"The interface looks not very attractive and does not look very modern."*<br>*"Looks like we have a lot of alerts to address."*<br>*"Now it's loading his chart"*<br>*"The black check mark—I'm looking back at the sheet—is a new organ system alert."*

		Descriptions of what is happening with a program (i.e. loading or freezing).<br>Participant summarizing information that is displayed on screen without reading words verbatim and without interpreting it. This includes describing vital signs or documentation, without an interpretation.<br>Participant summarizing outcomes of actions that they took.<br><u>Do not code</u><br>Reading verbatim from screen should be coded at Action Description.<br>Verbalization of a user's emotional response to the software programs should be coded at User Experience.<br>If a participant both summarizes the information and provides an interpretation, code at explanation.	*"There's a hemodynamic monitoring value that's out of range. There's a billing alert that I see, as well."*<br>*"Ok that did nothing."*<br>*"It looks like the patient was running about 115 over 60 with a heart rate in the 60s to 70s, up to 80s, to 20s-80s preop."*<br>*"You can see that they have treated the blood pressure."*
**EXPLANATION** [1-6]	Verbalization of <u>why</u> an action was chosen, either before, during, or after the action performed.<br><br>Statements that provide insight into motivation for behavior, or insight into internal thought processes.	<u>Code</u><br>Any explanation for why a course of action was chosen, including <u>why</u> specific buttons or programs were selected.<br>For AlertWatch alerts and features, include all statements related to alert evaluation, including whether the participant thinks an alert is relevant, what action they would recommend, and whether they would contact the OR provider. Also include what they are typing or selecting as they address alerts.<br>Include statements that describe *why* the participant would recommend a specific action to be taken.<br>Descriptions of theoretical actions that a participant might take in a situation related to the one that they are evaluating.<br>Interpretation of data that participant is evaluating.<br><u>Do not code</u>	"*I think I will look for a red alert since maybe those are more interesting*."<br>*"So I am going to now open Metavision because I don't really feel like I can comment on this case just knowing that the patient's hypotensive because what are their baseline MAPS?"*<br>*"Ok, I'm going to condense my time minutes here to quickly see the preop vital signs were along with the pre-induction and postinduction vital signs."*<br>*"I don't really see this [alert] as being significant"*<br>*"Regardless, the situation is resolved."*

		If a thought unit itself does not contain the explanation, code at Action Description, i.e. if a participant is simply stating out loud what they are selecting, without an explanation, code under Action Description.<br><br>If a participant is summarizing information on the screen without interpreting it, code at Observation.<br><br>If the participant's evaluation of an alert contains emotive content, code under user experience (i.e. "That's a very useful alert).	*"I would say that it is "potentially significant" – "would contact OR" – "consider decreasing the propofol infusion dose."*<br><br>*"So I am going to now open Metavision because I don't really feel like I can comment on this case"*
**USER EXPERIENCE**[2-6]	Descriptions of negative or positive emotions, feelings, or attitudes that arise during the testing sessions. Should reveal how participants experience the system.	Code<br><br>Explicit verbalization of emotions experienced by users (i.e. frustration, uncertainty, annoyance)<br><br>Verbalizations of how users perceive or describe the system (i.e. easy, difficult, confusing, interesting).<br><br>Reports of difficulty or ease of use of system, or hesitancy in using the system.<br><br>Include questions that participants verbalize during the session.<br><br>Indications of approval or disapproval of alerts, as long as emotive content is conveyed in thought unit.<br><br>Do not code<br><br>Descriptions of the system interface without an associated emotion or attitude should be coded under Observation.<br><br>Descriptions of the usefulness of individual alerts that do not contain emotive content should be coded under Explanation.	*"that's a good alert. That's a very useful alert."*<br><br>*"I'm not really sure where to start."*<br><br>*"I'm unclear as to what infusions 4.0 means – whether that means 4 different types of infusions? I don't know. I'm not sure what this means."*<br><br>*"so since I'm not entirely sure what's happening here, I'm also not entirely sure how Alert Watch can tell me a patient has a certain MAC of Propofol. That's quite interesting. I'm not sure that's a thing that exists."*<br><br>*"I have no idea where to look for anything."*<br><br>*"So in this case the horse is out of the bar so to speak and there's really no point in wasting your time with the alert"*
**REDESIGN PROPOSAL**[2-4]	Recommendations or suggestions to improve the system or to resolve difficulties that the participant	Code<br><br>Verbalizations of what participants think would make the software more useful, or what would make a specific alert more useful.	*"The useful thing in alert watch would be able to click on this and get a trend of blood pressures, like what the blood pressure was over the last 15 minutes."*

	encountered with the software or the setup.	Include process redesign proposals.<br>Include suggestions for revisions of existing alerts or for creation of new alerts.<br>Do not code<br>If participant is describing a difficulty with the system without a suggestion or recommendation for how it could be improved, code under user experience.	*"It would be good if the system would sort rather than by OR, rather than by the severity of the alert, so if it showed all of the red alerts on top, instead of the black alerts. That would guide you to be more efficient."*<br>*"Lack of train of four and MAC less than 0.7 would be a much more relevant alert"*<br>*"If someone could get a packet with your blood pressures, you've had instances like this multiple times you could send them those cases in an email and then also send them a link to an article that shows how this is associated with poor outcomes, something to be avoided."*
**DOMAIN KNOWLEDGE**[2, 3]	Knowledge or past experience with similar tasks or systems, including knowledge of the tested platforms.	Code<br>References to previous experiences with any of the software programs (Metavision, Clinical Desktop, AlertWatch), including prior knowledge of how to load programs or navigate within or between programs.	*"And instead of opening clindesk on the desktop I'm just going to try to get to it from the website since it's usually faster."*
**OTHER**	Verbalizations that do not fit into the remaining categories	Code<br>Brief verbalizations, such as individual words, filler-words, or phrases, that do not meet criteria for other categories.<br>Partial sentences for which the intent of the sentence cannot be clearly determined.<br>Includes participant talking to program/programs.<br>Do not code	*"Ok."*<br>*"There we go."*<br>*"Alrighty then."*<br>*"Obviously if the surgeon…" [thought not finished]*<br>*"In the meantime, I'm going to click back on the.."*

		If participant is talking to program and any sort of affective response is implicitly or explicitly stated, code at user experience.	
**Valence**[2]			
**POSITIVE**	Approval, satisfaction, or positive reactions to the individual components of the ACT or their integration	Code<br>Verbalizations conveying approval, satisfaction, and other positive reactions, either Implicitly and explicitly	*"Cumulative MAP 55 for 15 minutes—that's a good alert. That's a very useful alert."*<br>*"So it's good, an active alert."*
**NEGATIVE**	Disapproval, dissatisfaction, or negative reactions to the individual components of the ACT or their integration	Verbalizations conveying disapproval, dissatisfaction, and other negative reactions, either Implicitly and explicitly	*"I'm a little frustrated because right now it seems kind of a hassle to access all these programs to make a simple decision."*<br>*"and you can see in this case, the problem has been fixed for 20 minutes. This shouldn't be a red alert because the blood pressure is now fine."*<br>*"I don't really see this as significant unless they are planning on waking the patient up soon."*
**NEUTRAL**	Any comment neither positive nor negative	Descriptive statements without an associated valence.	*"Going to the web browser and opening up chrome."*<br>*"I am now looking over at alert watch looking for rooms that have potentially physiologic alerts."*

Table 2. Phase 1 ACT clinician usability testing: coding scheme for usability problem classification.

Topic	Definition
**Navigation**	Difficulty in navigating between programs or identifying necessary links or information

**Content**	Participants think that certain information is unnecessary or absent, or they have difficulty understanding information
**Functionality**	Participants encounter difficulties due to the absence of certain functions or the presence of problematic functions.
**Layout**	Participants encounter difficulties due to software display problems, e.g., visibility issues, inconsistencies.
*Modified from[3, 5]	

Table 3. Phase 3 Operating room clinician semistructured interview codebook.

**Domain**	Decision Rule and examples
**Domain as defined in TDF 1 and 2[7, 8]**	
**1. Behavioral Regulation** *Anything aimed at managing or changing objectively measured actions* **Michie[8]: What preparation is required? Are there procedures that encourage x?**	**Are there any existing strategies that influence implementation or utilization of ACT?**<br><br>**Consider coding to this domain:**<br><br>Descriptions of steps that would need to be taken to increase acceptance or utilization of the ACT support in routine clinical practice, either at a provider or an organizational level.
**2. Beliefs About Capabilities** *Acceptance of the truth, reality, or validity about an ability, talent or facility that a person can put to constructive use* **Michie: How difficult/easy is it to do x? What problems arise? What would help? How confident are people that they can do x despite**	**How confident are individuals in utilizing the ACT?**<br><br>Consider coding to this domain:<br><br>Descriptions of participant's confidence in being able to utilize or implement ACT support into routine clinical practice

**difficulties?**	
**3. Beliefs about Consequences**<br>*Acceptance of the truth, reality or validity about outcomes of a behavior in a given situation*<br><br>**Michie: What do they think will happen if they do or do not do x (to patients, themselves, the organization)? What are the costs of x and what are the consequences?**	**What are the negative or positive consequences associated with acceptance or utilization of ACT? What impact will it have on the individual provider, the department, or patient outcomes?**<br><br><u>Consider coding to this domain:</u><br><br>Beliefs about positive or negative outcomes from implementing ACT, including impact on:<br>• Work flow or work load<br>*"It seems that sometimes it may become an additional job to overwhelm me to have to explain to the control tower… that's more work for me."*<br>*"While on one hand the information might be useful; on the other hand it might be really difficult and distracting if you're trying to focus on the patient."*<br>• Professional satisfaction<br>*"I fear for the people outside and for the people who already work here would perceive it because…would it be hard to recruit?"* (double code at social influences)<br>*"But if it feels like it's something that just tying them up or micromanaging an otherwise very safe anesthetic, I think people would find that irritating."*<br>• Patient safety/outcomes<br>*"I think the way medicine is going, we have to think of novel and new ways to make sure that we take care of our patients…making sure that we have adequate care for all of our patients, so if this is something that we have to do to be more progressive and to move into the next generation, then I guess this is something we have to do."*<br>*"So I think there's a lot of situations where we can have that interaction to help improve patient care and safety because we're going to be covering a lot more rooms."*
**4. Emotion**<br>**Michie: Does doing x evoke an emotional response? To what extent do emotional factors facilitate or hinder x? How does emotion affect x?**	**Is there an emotional response to the ACT and if so, what is it and does it influence utilization or acceptance of ACT?**<br><br><u>Consider coding to this domain:</u><br>Descriptions of emotions experienced by clinicians in response to either specific case scenarios or general information, and how the emotions could impact implementation of the ACT<br>"*I think that would be a frustrating experience if it led to a lot more time communicating that either 'I'm working on it' or 'things are ok' or 'it's just an error.'"*

	Use for any **explicit** mention of an emotion (frustration, annoyance) that a scenario would evoke, and double code if needed for Beliefs about Consequences, Optimism/Pessimism<br>*"I think whenever your work is scrutinized, there's always that opportunity for a sense of resentment of that scrutiny…"*<br><u>Do not code</u><br>General opinions regarding benefits or drawbacks of ACT implementation should be coded at Optimism/Pessimism
**5. Environmental Context and Resources**<br>**Michie: To what extent do physical or resource factors facilitate or hinder x? Are there competing tasks and time constraints? Are the necessary resources available?**	**Is there anything in the CURRENT environment (physical constraints, etc) that facilitates or limits utilization or acceptance of ACT, or delivery of ACT support?**<br><br><u>Consider coding to this domain:</u><br><br>Discussion of anything in the <u>current</u> OR environment that would impact the utilization or usefulness of the ACT, including monitoring and equipment factors:<br>*"Because in the rooms, the new thermostats do not work at all, so I understand it's a huge problem. I'm not sure what I could do about it."*<br>*"Maybe you're A-line's dampened or something like that."*<br><u>Do not code:</u><br>If a resource is described or suggested that is NOT currently present, code under usability, "Suggestion"
**6. Goals & motivation/ intention**<br>*Mental representations of outcomes or end states that an individual wants to achieve*<br><br>**Michie: How much do they want to do x? How much do they feel they need to do x?**	**How much of a priority is it to utilize or implement ACT? What motivation is there to utilize ACT support?**<br><br><u>Consider coding to this domain:</u><br><br>Comments on how much of a priority it is to implement the ACT into routine practice.<br><br>Descriptions of the extent to which participants want to implement the ACT into routine practice
**7. Knowledge** *An awareness of the existence of something*	<u>**Are people aware of best clinical practices? Do they acknowledge awareness of alert content as recommended care? (focus on knowledge/cognitive awareness)**</u>

**From Michie:**<br>**What do people think the guidelines are or evidence is? Do people know why they should be carrying out certain behaviors?**	Consider Coding to this domain:<br><br>Awareness of current practice guidelines/best practice principles, including acknowledgment that alert content is representative of best practice.<br>*"[Train of four] has to be documented. We know that it's an alert…we have to check it, even if we gave succinylcholine and 10 [mg] of roc[uronium]."*<br><br>Participant's understanding of the perceived rationale behind the ACT or understanding of how it will function.<br>*"It seems pretty straightforward of how it will function. It seems like each, and correct me if I'm wrong, each patient for Alert Watch works…you dial in numbers if you will, and if they go out of that range, it's going to provide an error message. Now instead of having one person who has to, especially in the other operating rooms—not in Pod 3—flip through different screens or leave it up to that person's ability to catch those problems, I guess there will just be a fallback system if things are happening too…I don't think it's very complicated how it works."*<br><br>Descriptions of evidence that would convince participants to utilize the ACT support in routine practice.<br><br><u>Do not code</u><br>Descriptions of what a participant actually would do in a clinical situation, code under "Nature of Behavior"<br>Description of action that would be taken normally, regardless of alert, code under "Nature of Behavior"
**8. Memory, Attention and Decision Processes**<br>*The ability to retain information, focus selectively on aspects of the environment and choose between two or more alternatives*<br>**Michie: How much attention will they have to pay to do x? Will they remember to do x? Might they**	**In what situations would utilization be difficult? In what situations might people choose not to utilize ACT support and if so, why? Are there patient factors that would prevent an alert from being useful?**<br><br><u>Consider coding to this domain:</u><br><br>Description of **<u>situations in which utilization or delivery of ACT support would be difficult, due to:</u>**<br>• Competing tasks/time constraints<br>*"maybe you're not doing it because you're already overwhelmed with information that is really difficult to keep track of, that would just be another element of your confusion."* (triple coded with Emotion, Optimism/Pessimism)<br><br>• Patient or surgery/situation specific factors that impact the usefulness or applicability of alerts for

**decide not to do x (competing tasks, time constraints?)**	a given context:<br>*"is there something going on with the patient as far as their blood pressure control or other analgesic needs or other anesthetic requirements that might indicate that they need a higher MAC for their anesthetic; whether they have other higher narcotic requirements for the procedure."*<br>*"If I'm doing everything I can and it's still not improving, aside from the surgery finally ending, there's not a lot that I can do."*<br>*"if someone is a spine case, they're putting in lines, they're flipping, they're positioning and all that and then you're getting calls about something or pages about something, I think a lot of people would find that disruptive and not helpful."*<br>• Time constraints- refers to busyness, not urgency<br>*"You've got all this stuff to do and then you're literally pushing the antibiotic within 4 minutes of…time out…I really wouldn't want someone calling me."*<br>*"you get a lot of phone calls already, so if that's one of them…if you're busy, it's annoying. If you're not busy, it's an "okay, thank you" and then you move on."*<br>• Cognitive load<br><br>Also include situations in which utilization of the ACT would be easy:<br>*"This actually would be quite helpful because sometimes your set of eyes just don't catch that sometimes. It's just helpful when someone else says, "Hey, did you know this?" because sometimes you just miss stuff like that."*
**9. Nature of behavior**<br><br>**Michie: What is the proposed behavior? What do people currently do? How long are changes going to take? Are there systems for maintaining long term change?**	**What do participants currently/typically do, and what needs to be done differently? (Focus on action)**<br><br>Consider coding to this domain:<br><br>Comments on what participants already do in clinical practice, including how they would normally respond to a given clinical situation:<br>*"Personally, if I'm not redosing but I'm not yet preparing to emerge, I would certainly check train of four at the end. I wouldn't be making the situation worse because I'm not giving additional. On the other hand, I wouldn't want to extubate anyone that didn't have that documented. So I wouldn't see any great value in it at that point in time."*<br>*"I mean, generally, if we're all sitting there and the case is going fine, you're not too worried about that intubating dose, the case is still going on, you're not thinking about waking up, the patient seems fine, no one's complaining, therefore, that's not a priority of mine at that moment."*<br>*"It's not something that we typically put in."*

	Descriptions of actions that would be taken independent of Control Tower alert<br>Only code descriptions of <u>actions</u>; if they are describing that they simply agree with content of alert, or that they are aware of alert as best practice, code under **knowledge**
**10. Optimism**<br>*The confidence that things will happen for the best or that desired goals will be attained*	**What are the positive or negative attitudes regarding the ACT in terms of its overall effectiveness or impact on patient care or outcomes? What are the positive or negative attitudes towards individual alerts or scenarios?**<br><br><u>Consider coding to this domain:</u><br>Descriptions of optimism/pessimism towards the entirety of the ACT or its individual components:<br>*"I have a lot of impressions—favorable since I've agreed to participate in being one of the attendings in the ACT"*<br><br>Descriptions of whether participants think the ACT is useful in a specific scenario or for specific alerts, and what makes it useful or not useful:<br>*"I think just any time they can make a recommendation that is a practical recommendation, it would be helpful."*<br><br>Participant's descriptions of their optimism (or pessimism) regarding the potential impact of the ACT.<br>*"I don't know how much we're going to catch that will make a difference, that will truly make a difference."*<br><br>**Also can use as a general "valence" code; i.e. positive valence or negative valence. Mixed or neutral attitudes should also be coded here.**<br>Positive valence:<br>*Interviewer: Do you think it would be helpful in this situation; not helpful?*<br>*"I think it would be helpful."*<br>Negative valence:<br>*"Well, I think it's not much different than what my alarms do at this point, so I don't know how much of a benefit it is to have someone call me with a low blood pressure when that service is provided by the monitor…."*<br>Mixed valence:<br>"*While on one hand the information might be useful; on the other hand it might be really difficult and distracting if you're trying to focus on the patient."*

**11. Reinforcement**<br>*Increasing the probability of a response by arranging a dependent relationship, or contingency, between the response and a given stimulus*	**Are there rewards or potential for punishment for utilization of ACT support?**<br><br><u>Consider coding to this domain:</u><br><br>Any comments on rewards/reinforcement or punishment for utilization of ACT, including social reinforcement or punishment.<br><br>Descriptions of incentives to utilize ACT in routine practics

References

1. Cooke L. Assessing concurrent think-aloud protocol as a usability test method: A technical communication approach. IEEE Transactions on Professional Communication. 2010;53(3):202-15. DOI: 10.1109/TPC.2010.2052859.
2. Hertzum M, Borlund P, Kristoffersen KB. What do thinking-aloud participants say? A comparison of moderated and unmoderated usability sessions. International Journal of Human-Computer Interaction. 2015;31(9):557-70. DOI 10.1080/10447318.2015.1065691
3. McDonald S, Zhao T, Edwards HM. Dual verbal elicitation: the complementary use of concurrent and retrospective reporting within a usability test. International Journal of Human-Computer Interaction. 2013;29(10):647-60. DOI 10.1080/10447318.2012.758529.
4. Zhao T, McDonald S, editors. Keep talking: an analysis of participant utterances gathered using two concurrent think-aloud methods. Proceedings of the 6th Nordic Conference on Human-Computer Interaction: Extending Boundaries; 2010: ACM. DOI: 10.1145/1868914.1868979.
5. Zhao T, McDonald S, Edwards HM. The impact of two different think-aloud instructions in a usability test: a case of just following orders? Behaviour & Information Technology. 2014;33(2):163-83. DOI: 10.1080/0144929x.2012.708786.
6. Eveland Jr WP, Dunwoody S. Examining information processing on the World Wide Web using think aloud protocols. Media Psychology. 2000;2(3):219-44. DOI: 10.1207/S1532785XMEP0203_2.
7. Cane J, O'Connor D, Michie S. Validation of the theoretical domains framework for use in behaviour change and implementation research. Implementation science. 2012;7(1):37. DOI: 10.1186/1748-5908-7-37.
8. Michie S, Johnston M, Abraham C, Lawton R, Parker D, Walker A. Making psychological theory useful for implementing evidence based practice: a consensus approach. BMJ quality & safety. 2005;14(1):26-33. DOI: 10.1136/qshc.2004.011155.
