# Supplementary material for: Anesthesiology Control Tower—Feasibility Assessment to Support Translation (ACTFAST): Mixed-Methods Study of a Novel Telemedicine-Based Support System for the Operating Room
Source: JMIR Hum Factors. 2019 Apr 23;6(2):e12155. doi: 10.2196/12155 (PMC6658281; doi:10.2196/12155)
Supplement: Multimedia Appendix 3 [file humanfactors_v6i2e12155_app3.pdf]

Multimedia Appendix 3. Summary of changes to AlertWatch® Tower Mode platform.

Components of the Anesthesiology Control Tower (ACT), including the layout of the room, orientation documents, and the AlertWatch® Tower Mode platform, were modified and refined in an iterative fashion during the ACTFAST1 study based on results from Phase 1 and Phase 2 qualitative and quantitative data. Seven iterations of AlertWatch® Tower Mode were tested over the study period. Changes to the platform are summarized in Table 1; Figure 1 and Figure 2 demonstrate revisions to the software display.

Table 1. Description of changes in the 7 iterations of the AlertWatch® Tower Mode software.

**Version**	**Major features or changes**
1	First platform. ACT clinicians able to address checkmark alerts using free text (Figure 1A).
2	Introduction of limited pick lists for Assessment and Action (Figure 1B). Green checkmarks added to indicate completed/previously addressed alerts.
3	Pick list added for Reaction. Patient ID added to Case Review dialogue to help ensure that documentation is on correct patient.
4	Option added to dismiss an "irrelevant" alert for remainder of case. 17 checkmark alerts removed.
5	4 additional checkmark alerts removed. All alerts programmed to prevent from recurring for specific, individualized periods of time.
6	Significantly expanded and customized pick lists for assessment/action (Figure 1C). Enhanced Census View (Figure 2) with prioritization of alerts and additional case information.
7	Minor modifications to Census view.

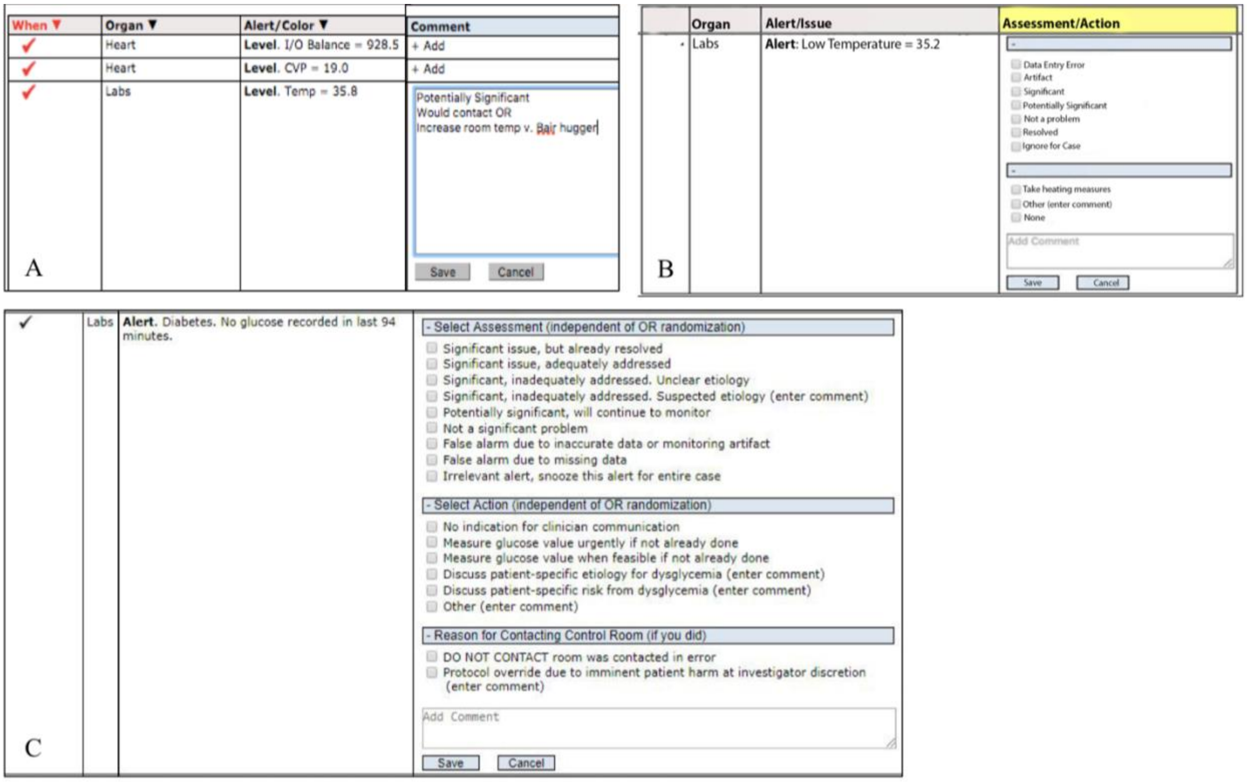

Figure 1. Changes to AlertWatch® Tower Mode Case Review Dialogue.

Figure 1A (top left). Clinicians in the ACT use the Case Review dialogue to address alerts generated by the AlertWatch® Tower Mode software. Full documentation includes the ACT clinician's assessment of the significance of the alert, what action they would recommend, and the response of the operating room clinician (for operating rooms in the experimental arm). The initial Case Review required participants to enter free text as they addressed individual alerts.

Figure 1B (top right). In the second iteration, a limited pick list was introduced that captured potential response.

Figure 1C (bottom). The final Case Review included an expanded pick list menu that was customized for each of the individual actionable alerts in the AlertWatch® Tower Mode program.

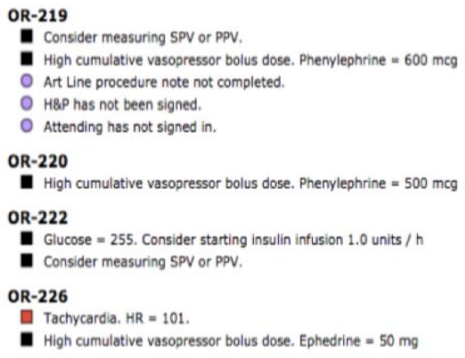

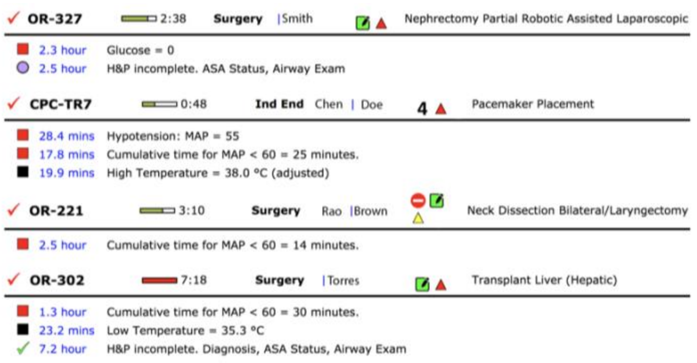

Figure 2A (left). **Original Census List View**

This view displayed active text alerts without any context, and was arranged only in order of operating room. Participants in Phase 1 had difficulty prioritizing alerts and stated a preference for assistance in determining alert severity.

Figure 2B (right). Modified Census List View

In the updated Census List view, introduced in the sixth iteration of the program, alerts are listed in order of priority, and additional information regarding the surgical procedure, assigned anesthesia providers, and duration of surgery is provided. Alerts that were previously addressed by the ACT are indicated by green checkmarks.
