# Supplementary material for: Anesthesiology Control Tower—Feasibility Assessment to Support Translation (ACTFAST): Mixed-Methods Study of a Novel Telemedicine-Based Support System for the Operating Room
Source: JMIR Hum Factors. 2019 Apr 23;6(2):e12155. doi: 10.2196/12155 (PMC6658281; doi:10.2196/12155)
Supplement: Multimedia Appendix 4 [file humanfactors_v6i2e12155_app4.pdf]

Multimedia Appendix 5: Redesign proposals.

Redesign proposals for the Anesthesiology Control Tower were gathered from the Phase 1 think-aloud and debriefing sessions.

Table 1. Redesign proposals as related to usability problem categories and themes identified in the Phase 1 ACT clinician think-aloud and debriefing sessions.

Category[1]	Theme	Number of participants reporting	Examples
Navigation	Suggestions for easier way to finding link or information	1	*"It would be better if there was a folder with direct hyperlinks rather than a [help] document."* 2102, attending physician
	Any Navigation proposal	1	
Content	Improved meaning or relevance of alerts	7	*"[A] more useful alert would be the time period, if there was a high dose of vasopressor given recently rather than cumulatively. Like over the last 20 minutes or something."*2102, attending physician
	Enhanced prioritization of alerts	5	"*But I wish that there was a way that it could prioritize the different alerts so even if you have 10 rooms that have alerts that need to be interrogated, you know these are the top 3 that I need to focus on right now versus the other ones."* 2111, resident physician
	Increased availability of relevant information	3	*"Otherwise I would like a legend that tells you everything about what the alert possibly means."* 2105, resident physician
	Suggestions for identifying correct patient or operating room	1	*"Wouldn't it be nice if, when I see the window, the ACTFAST case review, it had the patient's name right there so I could be sure I was addressing the correct patient?"* 2112, resident physician
	Any content proposal	10	
Functionality	Improved interoperability of software programs	4	*"Maybe it would be nice if you click on an OR in Alert Watch it would automatically open in [the anesthesia information management system] and [the electronic health record], but I feel like that's too much to ask for."* 2115, attending physician

	Any Functionality proposal	4	
Layout	Increased visibility of text	3	*"[It] would be useful if this could be seen, the whole case [name] somehow."* 2102, attending physician
	Improved physical layout (monitors)	1	*"I think it would be more efficient if the attending had another screen and then this screen was a separate computer that anyone could pull up – that way if the resident wanted to show me something I could still be doing my work and he could pull up the alert watch on that screen and show everybody."* 2102, attending physician
	Any Layout proposal	4	

References

1. Zhao T, McDonald S, Edwards HM. The impact of two different think-aloud instructions in a usability test: a case of just following orders? Behaviour & Information Technology. 2014;33(2):163-83. PubMed PMID: 10.1080/0144929X.2012.708786.
