# Supplementary material for: Anesthesiology Control Tower—Feasibility Assessment to Support Translation (ACTFAST): Mixed-Methods Study of a Novel Telemedicine-Based Support System for the Operating Room
Source: JMIR Hum Factors. 2019 Apr 23;6(2):e12155. doi: 10.2196/12155 (PMC6658281; doi:10.2196/12155)
Supplement: Multimedia Appendix 5 [file humanfactors_v6i2e12155_app5.pdf]

Multimedia Appendix 5. Summary of relevant belief statements and sample quotes from attending anesthesiologists and certified registered nurse anesthetists (CRNAs). In these sessions participants were invited to provide their feedback on the ACT in general and on six specific clinical scenarios in which they imagined themselves receiving ACT support.

Domain	Specific belief, Emotion, or Issue Identified	Sample quote	Number reporting (N=10)
Beliefs about capabilities	Not confident in one's ability to interact with the ACT	"I'm not even sure how easy it's going to be to communicate. I don't know what channels we are going to use" 2120, attending physician	1
Beliefs about consequences	*Benefits of ACT implementation*		
	Improved patient outcomes	"Just like anything else, we'll get used to it and do the best for the patients, right?" 2124, CRNA	3
	Advancement of patient care and field of anesthesia	"I applaud you guys for working on this and trying to find something that's a novel and efficient and exemplary way of trying to take care of patients as a team." 2116, attending physician	3
	Disadvantages of ACT implementation		
	Worsened patient outcomes	"You have someone in the [ACT] who is recommending that you take steps on the anesthetic care of a patient, and if it's a recommendation that is not appropriate, that would be really unfortunate." 2118, CRNA	2
	Distraction from patient care	"While on one hand the information might be useful; on the other hand it might be really difficult and distracting if you're trying to focus on the patient." 2118, CRNA	4
	Workload would increase	"It may become an additional job… to have to explain to the control tower…and give [the control tower] the background that they don't have so that they can work better." 2122, attending physician	3

	Professional satisfaction would be decreased	"So one problem I guess I'm talking about is just the sense of independence when you're working, and if that's reduced, does that take away from the satisfaction of the job?" 2125, CRNA	4
Emotion	Annoyance or Frustration	"And then some of the other more low priority things may get a little annoying, I guess…you get a lot of phone calls already …if you're busy, it's annoying." 2121, CRNA	7
	Embarrassment	"If I'm not working on it, I probably feel embarrassed to be honest, unless it's some sort of error in the system." 2121, CRNA	2
	Worry	"It's also scary in a way for some of us. It's a little bit of a "Big Brother" feeling." 2120, CRNA	3
Environmental context and resources	Limitations with equipment and monitors would decrease ACT usefulness	"I think with our monitoring devices that we have for [depth of pharmacologic paralysis], sometimes it makes it a little bit more difficult to make sure [a patient has recovered]. 2116, attending physician	3
	Limitations with communication devices would decrease ACT usefulness	"Well, our pager system is highly unreliable…my pager is set up so that I also get a message sent to my phone and it's not uncommon for me to get it only on my phone and I never get it on my pager. Especially around lunch time, it can be a 30 minute delay before you get a page." 2118, CRNA	4
Knowledge	I am aware of/agree with the practices discussed in the clinical scenarios.	"The [depth of pharmacologic paralysis] certainly is important to document and important to make sure that the patient has recovered from their neuromuscular blockade." 2116, attending physician	7
	I understand the rationale behind ACT/how ACT would function	"Well, going through these scenarios now it makes more sense, having a picture of what they can do. It's definitely one of the ways how to improve our work." 2117, attending physician	7

	I would need more evidence to support routine use of the ACT	"Because we could find out that there is so much…there's a bigger problem than what we thought…that would be shocking to me, and it would be good to know, too, but it would be shocking that we would have such huge variances in how we're all practicing as far as safety, major safety areas…So it will be interesting to see what the final data is." 2121, CRNA	1
Memory Attention Decision processes	Patient or case specific factors could limit usefulness of ACT	"It depends on the context, what I already know about the patient and what I know that the control tower may know or not know about the patient." 2122, attending physician	8
	Patient or case specific factors could enhance usefulness of the ACT	"I think it's very helpful …if you have type 1, a type 1 [diabetic] patient …a patient that…really needs management" 2121, CRNA	5
	Workload, including cognitive workload, would benefit from ACT implementation	"I think maybe bouncing off what I've done… it's always nice to have communication and another thought about what you can do to help remedy the situation." 2119, CRNA	5
	Workload, including cognitive workload, would suffer from ACT implementation	"If something's actually happening and you're actually providing patient care to try to alleviate that problem and your phone's ringing and ringing and ringing, it's just not a nice situation." 2119, CRNA<br><br>"I'm still a little bit worried about the distraction point—how much would I have to take of my already very limited, very stretched time to get back to the control tower and give them more context that they might need to be of more help to me." 2122, attending physician	6
	Timing could negatively impact ACT usefulness	"Timing, I think, is important. And if someone is a spine case, they're putting in lines, they're flipping, they're positioning and all that, and then you're getting calls about something or pages about something, I think a lot of people	5

		would find that disruptive and not helpful." 2118, CRNA<br><br>"it's going to interrupt me a little bit…that would be maybe an issue." 2121, CRNA	
	Timing could positively impact ACT usefulness	"If that person [in the ACT] has information the [clinicians in the OR] really need right then, it would be more timely to have it sooner than later if that makes sense." 2118, CRNA	1
Nature of Behavior	ACT alert supports provider's current practice patterns	"Since I work 12 hours, I relieve a lot of people, and a lot of rooms I go into, I [already] do turn the [anesthetic] agent down when I go in just because a lot of the machines don't do it for you automatically." 2119, CRNA	
	ACT alert contradicts provider's current practice patterns	"I mean, generally, if we're all sitting there and the case is going fine, you're not too worried about that intubating dose [of neuromuscular blocking agent]… Can I check [depth of pharmacologic paralysis]? Sure, but it's probably not as useful information." 2121, CRNA	
Optimism and pessimism	Optimism towards ACT in general or towards specific alerts	"It's always nice to have someone else kind of watching out on a situation for you. Just like the [Intensive Care Unit] upstairs. I don't really think that there's anything that you can say that's really bad about the system." 2119, CRNA<br><br>"I think who this is really helpful for is for some of our less experienced people who maybe don't know they're in trouble until they're *really* in trouble, and then they really need help." 2121, CRNA	10
	Pessimism towards ACT in general or towards specific alerts	"Yes, my initial impression is that I am not entirely convinced of the value of the control tower in our setting. I wish that there were other ways that we could improve our adherence to good practices and reduce our rate of errors and not	9

		having to be controlled by a control tower.” 2122, attending physician	
	Mixed reaction towards ACT in general or towards specific alerts	“It would be helpful depending on the situation…or it could be a distractor in this case.” 2122, attending physician<br><br>“I guess depending on the timing of the receipt of that hint or reminder, it could be either perceived as very helpful or somewhat of a nuisance.” 2125, CRNA	9
Social influences	Opinion of colleagues would limit ACT usefulness	“I think the reality is that there’s going to be a number of people who are just plain offended by the thought of someone looking over their shoulder while they practice.” 2125, CRNA	5
	Opinion of colleagues would enhance ACT usefulness	“I think everyone is committed to patient safety and the best patient care possible, and if this is deemed to be a legitimate tool that helps with that, I think it would be welcome.” 2118, CRNA	2
Social Professional Role and Identity	ACT supports current structure for anesthesia practice	“So I think there’s a lot of situations where we can have that interaction to help improve patient care and safety because we’re going to be covering a lot more rooms.” 2116, attending physician	1
	ACT conflicts with current structure for anesthesia practice	“If the person who is in the tower is making the recommendation, do they in any way have a legal responsibility to that patient? Are they signing on as some advisor to that medical record?” 2118, CRNA	1
	ACT support is consistent with current professional norms	“Ultimately someone who is having a picture of all of [the reminders] may be definitely also helpful, especially…if we are covering three rooms. If [things] are very busy, patients are difficult, and physically sometimes you can’t really cover all minor mistakes…so it may be helpful.” 2117, attending physician	5
	ACT support conflicts with current professional norms	“My first question is why do we need it because if we have all the resources…if we are professionals, if we are taking good care of the patient, why do we still	4

		need someone to supervise us?” 2117, attending physician<br><br>“Why are the providers not considered sufficient, and is there any way that we can improve their performance if we perceive that they need support in their performance?” 2122, attending physician	
	ACT would negatively impact professional autonomy	“People want to come and work here because they get to do things they don’t get to do all the time. They develop a lot of autonomy and it’s fun for them. The downside is someone directing every second of your day.” 2121, CRNA	5
Domains determined to be irrelevant (no participant voiced belief statement in category): behavioral regulation, goals and motivation, reinforcement, and skills			
